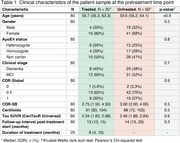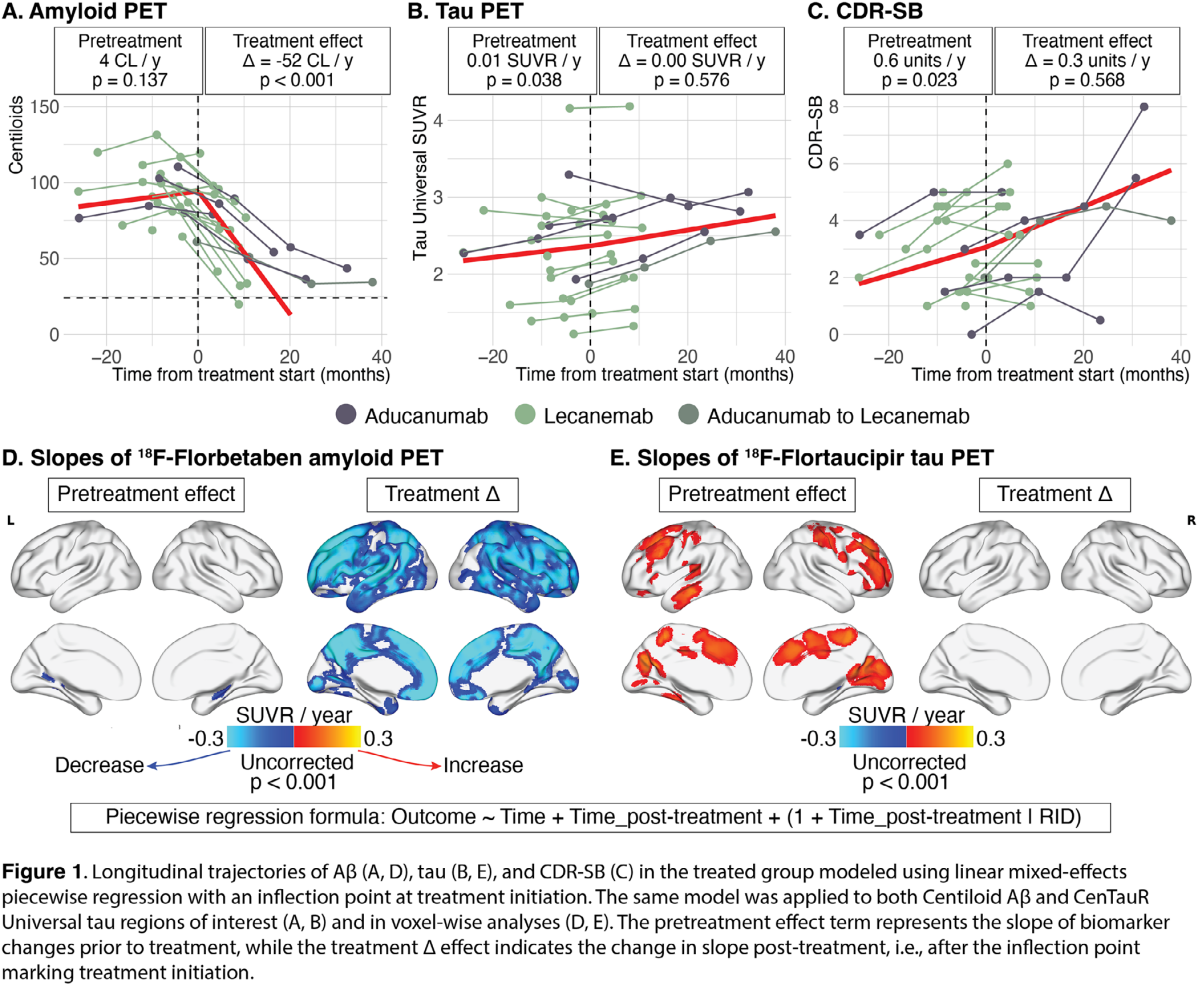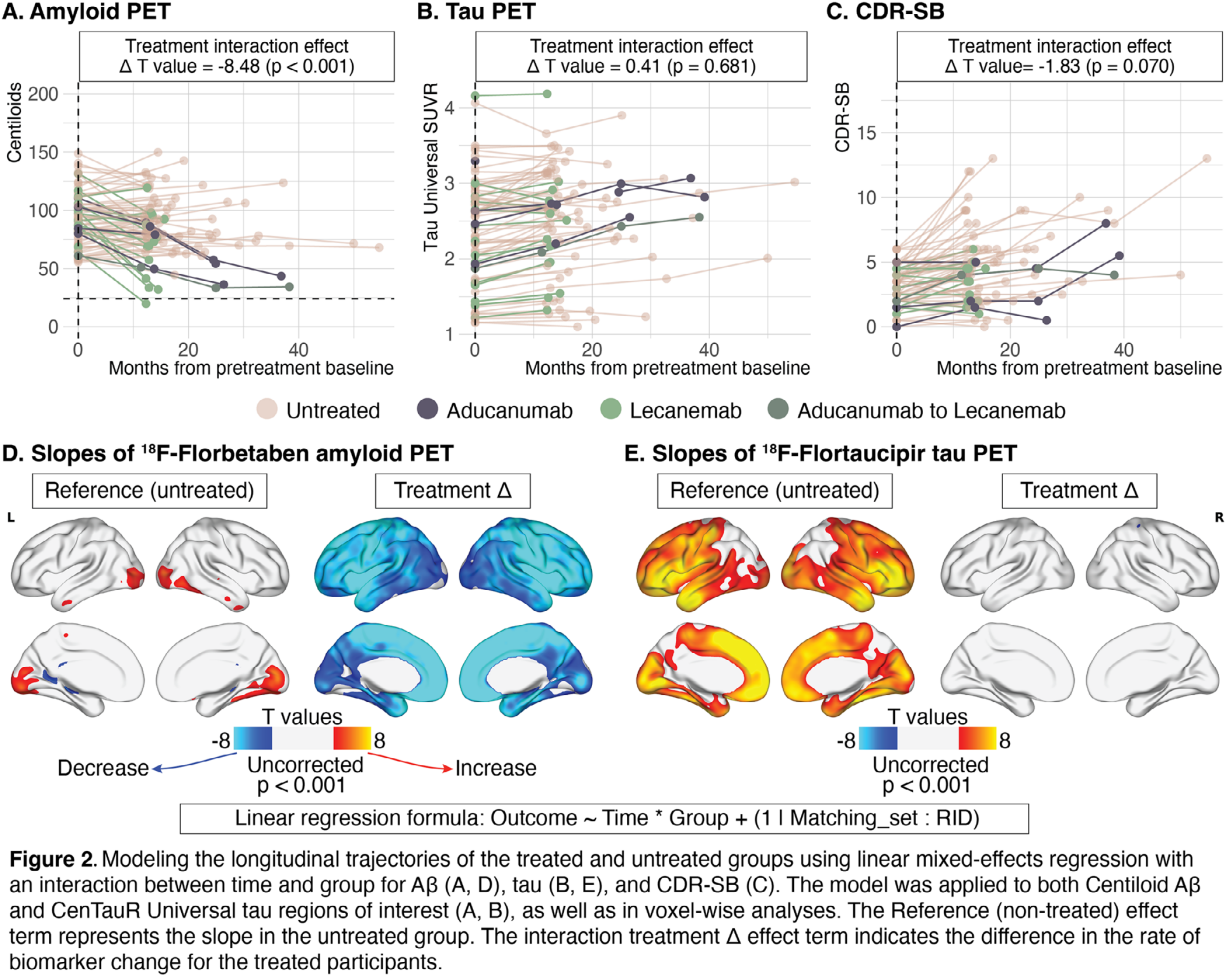# Target Engagement and Cognitive Outcomes of Anti‐Amyloid Immunotherapies in Early‐Onset Alzheimer’s Disease: First Report from the LEADS Study

**DOI:** 10.1002/alz70862_110255

**Published:** 2025-12-23

**Authors:** Konstantinos Chiotis, Ganna Blazhenets, Ani Eloyan, Piyush Maiti, Jiaxiuxiu Zhang, Alexandra Touroutoglou, Kala Kirby, Dustin B. Hammers, Maria C. Carrillo, Brad C. Dickerson, Liana G. Apostolova, Gil D. Rabinovici

**Affiliations:** ^1^ Memory and Aging Center, Weill Institute for Neurosciences, University of California San Francisco, San Francisco, CA USA; ^2^ Department of Biostatistics, Brown University, Providence, RI USA; ^3^ Frontotemporal Disorders Unit and Massachusetts Alzheimer’s Disease Research Center, Department of Neurology, Massachusetts General Hospital and Harvard Medical School, Boston, MA USA; ^4^ Indiana University School of Medicine, Indianapolis, IN USA; ^5^ Alzheimer's Association, Chicago, IL USA; ^6^ Department of Neurology, Indiana University School of Medicine, Indianapolis, IN USA; ^7^ Department of Medical and Molecular Genetics, Indiana University School of Medicine, Indianapolis, IN USA; ^8^ Department of Radiology and Imaging Sciences, Center for Neuroimaging, Indiana University School of Medicine, Indianapolis, IN USA; ^9^ Department of Radiology and Biomedical Imaging, University of California San Francisco, San Francisco, CA USA

## Abstract

**Background:**

In clinical trials, monoclonal antibodies targeting Aβ pathology have shown strong target engagement, resulting in rapid Aβ clearance and a deceleration in rate of clinical decline. Now that these treatments are approved and implemented in clinical practice, we could assess their effects in observational studies involving these patients.

**Method:**

We analyzed data from 20 participants with early‐onset Alzheimer’s disease (EOAD) in the Longitudinal Early‐Onset Alzheimer’s Disease Study (LEADS) cohort, treated with Aducanumab (*n* = 4), Lecanemab (*n* = 15), or both (one transitioning from Aducanumab to Lecanemab). All participants had MCI or mild dementia at baseline, longitudinal Aβ and tau PET, as well as cognitive assessments, with at least one observation before and after treatment initiation. We applied piecewise regression with a knot at the treatment start, to evaluate changes in Aβ and tau PET burden and Clinical Dementia Rating‐Sum of Boxes (CDR‐SB) scores. We compared the trajectories of treated participants with an untreated group (i.e., treated‐untreated comparison) from LEADS, matched for age, sex, APOE ε4 genotype, pretreatment Aβ and tau PET load, CDR‐SB, and follow‐up duration, using a 1:3 matching design.

**Result:**

The median treatment duration was 8 months (IQR=5‐10). In the piecewise regression model, the treated group showed significant decreases in Aβ burden post‐treatment (Δ=‐52 Centiloids/yr, *p* <0.001) with widespread neocortical involvement (Figure 1). However, no significant inflection in tau burden (Δ=0 SUVR/yr, *p* = 0.58) or CDR‐SB (Δ=0.3 units/yr, *p* = 0.57) trajectories was observed. In the treated‐untreated comparison, the treated group showed a trend toward slower increases in CDR‐SB scores post‐treatment (ΔT=‐1.8, *p* = 0.07) compared to the untreated group (Figure 2). Aβ levels significantly decreased in the treated group compared to the untreated group (ΔΤ=‐8.5, *p* <0.001). No significant differences in tau trajectories were observed between groups (ΔT=0.4, *p* = 0.68), with both showing increases in cortical regions of interest.

**Conclusion:**

We observed excellent target engagement, with piecewise regression models showing rates of Aβ clearance comparable to those seen in Phase 3 trials. The study was underpowered to detect cognitive benefits, which are limited over a short follow‐up interval. These findings underscore the utility of observational studies with biomarker data in evaluating treatment efficacy, offering insights that complement traditional randomized trials.